# Antimicrobial susceptibility pattern of clinical isolates from cases of ear infection using amoxicillin and cefepime

**DOI:** 10.1186/2193-1801-2-288

**Published:** 2013-07-01

**Authors:** Shaheen Perveen, Syed Baqir Naqvi, Anab Fatima

**Affiliations:** Department of Pharmaceutics, Faculty of Pharmacy, University of Karachi, Karachi, Pakistan; Department of Pharmaceutics, Faculty of Pharmacy, Jinnah University for Women, Karachi, Pakistan; Department of Pharmaceutics, Faculty of Pharmacy, Hamdard University, Karachi, Pakistan

**Keywords:** Otitis media, Resistant, Pseudomonas aeruginosa, Staphylococcus aureus

## Abstract

The aim of the present study was to determine the sensitivity pattern of clinical isolates of otitis media. During the last few decades, the occurrence of otitis media seems to have been rising probably because of prevalence of multidrug-resistant *Pseudomonas aeruginosa* and β-lactamase producing *Staphylococcus aureus* in the pathogenesis of otitis media. *Pseudomonas aeruginosa* and *Staphylococcus aureus* were the most common causative microorganisms of ear infection. Keeping in view the importance of these pathogens, the present study had been designed to determine the sensitivity pattern of clinical isolates of otitis media. These isolates were collected from different hospitals and pathological laboratories of Karachi and their sensitivity against cefepime and amoxicillin were determined by using disk diffusion method. The results have shown that *Pseudomonas aeruginosa* was the most common causative microorganism of ear infection. Cefepime, a fourth generation cephalosporin appeared to be an effective antibiotic against *Pseudomonas aeruginosa* and *Staphylococcus aureus*.

## Introduction

The term Otitis media (OM) generally used to describe any inflammatory process involving the middle ear. The main categories of OM are 1) Acute Otitis Media (AOM), 2) Secretory otitis media (SOM), also named chronic otitis media with effusion (COME) and 3) Chronic otitis media (COM) with or without cholesteatoma (Harkness and Topham 1998). Acute Otitis Media is a condition with acute middle ear effusion (MEE) and acute signs and symptoms of infection for example fever, pain, restless sleep, irritability, tugging or rubbing of ears. AOM may also be accompanied with acute discharge from the ear. Recurrent AOM is defined as a condition with at least three AOM episodes in 6 months or 4 episodes within a year (Klein 1984; Alho 1997). Secretory otitis media SOM (or COME) means that MEE has lasted at least 2–3 months behind intact tympanic membrane. A permanent perforation of tympanic membrane with recurrent or constant purulent discharge indicates COM. This can be associated with cholesteatoma, a condition where keratinizing stratified squamous epithelium accumulates in the middle ear cleft (Pellman 1999). Chronic suppurative otitis media (CSOM) involves a perforation (hole) in the tympanic membrane and active bacterial infection within the middle ear space for several weeks or more. Otitis media is very important because it is the earliest, or the first infection that occurs to children. Statistics showed 80-90% of children have at least one episode of otitis media by the age of 2 years (Boston 1989). From the pathogenic point of view, the nasopharyngeal carriership of pathogenic bacteria was a risk factor for AOM (Harabuchi et al. 1994Faden et al. 1997), and as it has been shown that the same bacteria that have been identified in the middle ear also found in the nasopharynx. Loos et al. (1989), it was a rational to assume that bacteria enter the middle ear from the nasopharynx through the Eustachian tube. One of the most significant pathogenic factor in the development of otitis media was the dysfunction of the Eustachian tube. Bluestone (1974), showed that young children have shorter, straighter and more compliant Eustachian tube than adults. This permits a reflux from the nasopharynx to the middle ear with the consequence of bacterial contamination (Hooton 2000).

The improper and indiscriminate use of antimicrobials in our country regarded as a leading cause for higher percentage of resistant bacteria isolated from middle ear effusions of children.

The main objective of the present study was to study the susceptibility patterns of clinical isolates causing ear infection.

## Material and method

In the present study, susceptibility patterns of clinical isolates from ear infection were determined. For this purpose, two hundred clinical isolates of ear infection were collected over a period of six months. Antibiotics discs were purchased from Oxoid.

The clinical isolates of *Staphylococcus aureus* and *Pseudomonas aeruginosa* from ear infection were collected from different hospitals and laboratories including NICH, Ziauddin hospital, Dr. Essa’s lab, Sind Lab and Mehdi Manji Lab. The culture swabs were collected at weekly intervals and safely transported to microbiology laboratory then the organisms were isolated in pure form and identified on the basis of characters given in Bergey’s manual. Then pure isolates of *Pseudomonas aeruginosa* were transferred to 1% nutrient agar slant and were stored in the refrigerator at 4 ±1°C. Different identification tests were performed on suspected *Pseudomonas aeruginosa* and were characterized and identified. Similarly verification tests for Gram-positive *Staphylococcus aureus* were also performed for pure isolation. The antibiotics used were Cefepime, a 4^th^ generation cephalosporin and Amoxicillin, a semi-synthetic penicillin.

### Disk diffusion method

Antimicrobial agent susceptibilities were determined by disk diffusion method. The method used was described in the NCCLS (2000) guidelines using Mueller-Hinton agar and broth (Oxoid, UK). The medium containing antimicrobial agent disk were quality controlled daily. The plates were examined after specified incubation and then the zone of inhibition were measured and then compared with NCCLS susceptibility of *Staphylococcus aureus* and *Pseudomonas aeruginosa.*

### Interpretation

The diameters of the zone of inhibition around each disk were measured with a vernier caliper and were interpreted as sensitive or resistant according to the zone interpretive standards.

## Results

The newer fourth generation cephalosporin, cefepime is effective against multidrug -resistant *Pseudomonas aeruginosa* and β-lactamase producing strains of *Staphylococcus aureus.*

**Figure 1:**
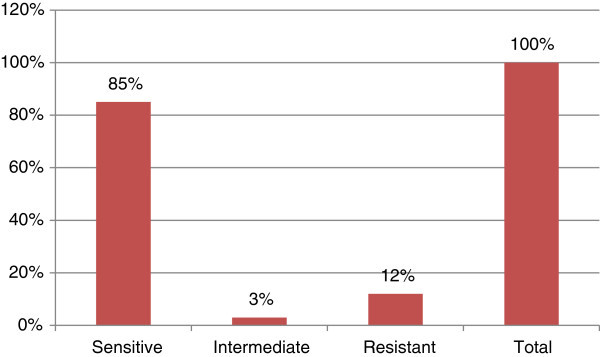
**Susceptibility pattern of*Staphylococcus aureus*to Cefepime.**

**Figure 2:**
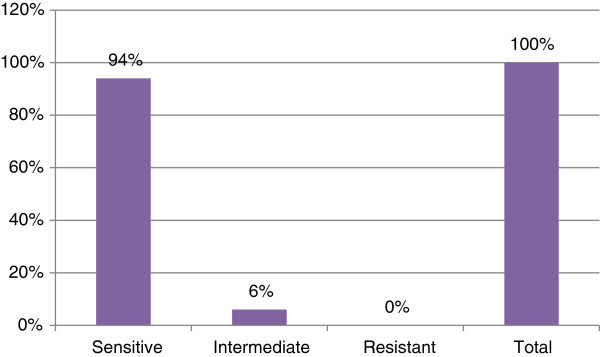
**Susceptibility pattern of*Pseudomonas aeruginosa*to Cefepime.**

**Figure 3:**
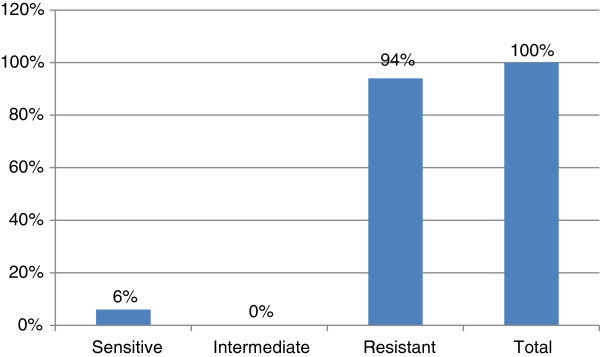
**Susceptibility pattern of*Staphylococcus aureus*to Amoxicillin.**

**Figure 4:**
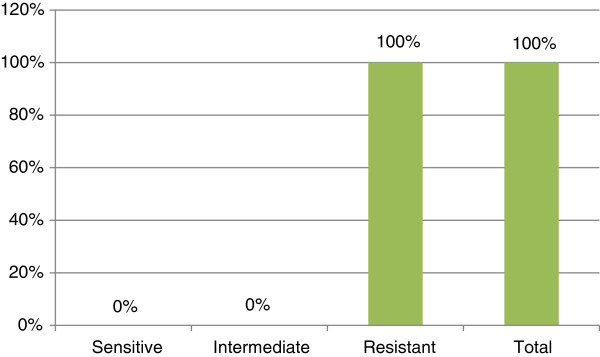
**Susceptibility pattern of*Pseudomonas aeruginosa*to Amoxicillin.**

Thus, cefepime is an alternative for the management of acute otitis media. Antibiotic susceptibilities of clinical isolates from ear infection were summarized in Figures 1, 2, 3 and 4. Cefepime showed 85% sensitivity against *Staphylococcus aureus* as shown in Figure 1. Cefepime is the most effective antibiotic against *Pseudomonas aeruginosa* having 94% sensitivity as shown in Figure 2.

## Discussion

Antibiotic resistance to available microorganisms has been constant since the introduction of sulfonamides in the 1930s. Multidrug resistant *Pseudomonas aeruginosa* and β-lactamase producing *Staphylococcus aureus* become a matter of great concern now because of the importance of these pathogens in middle ear infection of infants and children.

The risk factors for the development of resistance in otitis media include improper and indiscriminate use of antimicrobial, young age, tobacco smoke, male gender, day-care attendance and immunodeficiency. The understanding of the pathogenesis of acute otitis media (AOM) was the basis of discovering effective strategies in the management of this disease. According to Lim et al. in 2000and the sterility become maintained by the mucociliary system and by the enzymes and antibodies secreted by the epithelial cells of the Eustachian tube and the middle ear. Henderson et al. in 1982 and Heikkinen et al. in 1995 reported that although AOM is usually a bacterial infection but often proceeded by a respiratory viral infection. The same otitis pathogens that were found in middle ear effusion also found in the nasopharynx (Loos et al. 1989). Although AOM can occur at any age, it is most common in young children, particularly at the age of 6–24 months (Pukander et al 1982; Lundgren and Ingvarsson 1983; Teele et al. 1989). A genetic component engrossed in the predisposition to middle ear infections (Casselbrant and Mandel 2001).

Cefepime, the fourth generation cephalosporin has provided a new therapeutic possibility, offering a wide range of antibacterial activity and proven concentration in the middle ear.

Amoxicillin also used for treatment of otitis media but its major drawback was limited efficacy when β-lactamase producing bacteria were the major causative microorganisms. *Klebsiella pneumoniae* and *Bacteroide spp*. rarely caused acute otitis media (Robert 1992). Despite the availability of many other drugs, amoxicillin is still the drug of first choice in most cases (Klein 1994; McCracken 1994). In 1989, Brook and Yocum found that in suppurative otitis media, *Pseudomonas aeruginosa*, *Klebsiella pneumoniae* and *Staphylococcus aureus* were the most common bacteria. Mozafari Nia et al. in 2011 reported that the *Staphylococcus aureus* was the commonest aerobic isolate in CSOM. *Pseudomonas* isolates also showed complete (100%) resistance to amoxicillin/clavulanate. Our findings also correlate with Mozafari findings.

In 2002, AHC Loy et al. found that the most common causative organism of chronic suppurative otitis media are *Pseudomonas aeruginosa* (33.3%) and *Staphylococcus aureus* (33.3%) followed by coagulase negative *Staphylococcus aureus* (21.1%). Oni et al. (2001) reported that *Pseudomonas aeruginosa* was the predominant agent of chronic supprative otitis media and acute supprative otitis media. This was followed by *Staphylococcus aureus*. Kenna et al. (1986) reported that in chronic suppurative otitis media, the most common organisms are *Pseudomonas aeruginosa* and *Staphylococcus aureus*. According to Aslam et al. (2004) The commonest microorganisms isolated from chronic discharging ears were Pseudomonas aeruginosa and Staphylococcus aureus. This was also confirmed from our findings.

Among the cephalosporins, cefepime was used in the present study. All isolates of *Pseudomonas aeruginosa* were resistant to cefepime while only 12% isolates of *Staphylococcus aureus* (were resistant to cefepime. Ale Zehra et al. 2010) also found cefepime effective against *Pseudomonas aeruginosa*. *Pseudomonas aeruginosa* and *Staphylococcus aureus* are the most frequently isolated pathogens in patients with acute otitis media. The recent emergence of multidrug-resistant *Pseudomonas aeruginosa* and increasing frequency of β-lactamase producing strains of *Staphylococcus aureus* are creating problem regarding the use of amoxicillin as first line empiric therapy for acute otitis media in young children. The new fourth generation cephalosporin, cefepime is effective against multidrug -resistant *Pseudomonas aeruginosa* and β-lactamase producing strains of *Staphylococcus aureus*.

## Conclusion

Thus, Cefepime is an alternative for the management of otitis media. Hence the present study would be helpful for the Doctors and the Pharmacists in prescribing the medicine to the patients suffering from otitis media.
